# Aligning the Measurement of Microbial Diversity with Macroecological Theory

**DOI:** 10.3389/fmicb.2016.01487

**Published:** 2016-09-23

**Authors:** James C. Stegen, Allen H. Hurlbert, Ben Bond-Lamberty, Xingyuan Chen, Carolyn G. Anderson, Rosalie K. Chu, Francisco Dini-Andreote, Sarah J. Fansler, Nancy J. Hess, Malak Tfaily

**Affiliations:** ^1^Pacific Northwest National Laboratory, Biological Sciences DivisionRichland, WA, USA; ^2^Biology Department and Curriculum in Environment and Ecology, University of North CarolinaChapel Hill, NC, USA; ^3^Pacific Northwest National Laboratory, Joint Global Change Research InstituteCollege Park, MD, USA; ^4^Pacific Northwest National Laboratory, Atmospheric Sciences and Global Change DivisionRichland, WA, USA; ^5^Pacific Northwest National Laboratory, Environmental Molecular Sciences LaboratoryRichland, WA, USA; ^6^Microbial Ecology Cluster, Genomics Research in Ecology and Evolution in Nature (GREEN), Groningen Institute for Evolutionary Life Sciences (GELIFES), University of GroningenGroningen, Netherlands

**Keywords:** species richness, OTU richness, species energy theory, niche conservatism, rarefaction, soil, permafrost, boreal forest

## Abstract

The number of microbial operational taxonomic units (OTUs) within a community is akin to species richness within plant/animal (“macrobial”) systems. A large literature documents OTU richness patterns, drawing comparisons to macrobial theory. There is, however, an unrecognized fundamental disconnect between OTU richness and macrobial theory: OTU richness is commonly estimated on a per-individual basis, while macrobial richness is estimated per-area. Furthermore, the range or extent of sampled environmental conditions can strongly influence a study's outcomes and conclusions, but this is not commonly addressed when studying OTU richness. Here we (*i*) propose a new sampling approach that estimates OTU richness per-mass of soil, which results in strong support for species energy theory, (*ii*) use data reduction to show how support for niche conservatism emerges when sampling across a restricted range of environmental conditions, and (*iii*) show how additional insights into drivers of OTU richness can be generated by combining different sampling methods while simultaneously considering patterns that emerge by restricting the range of environmental conditions. We propose that a more rigorous connection between microbial ecology and macrobial theory can be facilitated by exploring how changes in OTU richness units and environmental extent influence outcomes of data analysis. While fundamental differences between microbial and macrobial systems persist (e.g., species concepts), we suggest that closer attention to units and scale provide tangible and immediate improvements to our understanding of the processes governing OTU richness and how those processes relate to drivers of macrobial species richness.

## Introduction

A large body of literature in plant and animal ecology (“macrobial ecology”) has focused on spatial gradients in species richness, where researchers have proposed myriad hypotheses to explain observed patterns (Rohde, 1992; Willig et al., 2003; Currie et al., 2004). Recent efforts have focused on hypotheses related to resource supply, evolutionary time, phylogenetic niche conservatism, and environmental temperature (Hurlbert and Stegen, 2014). While advances in molecular tools have facilitated the transfer of theory from macrobial ecology into microbiology (e.g., Hanson et al., 2012), relatively few studies have tested these hypotheses in microbial systems (e.g., Bienhold et al., 2012).

A greater emphasis—within microbial ecology—on evaluating and extending macrobial species richness hypotheses would likely be beneficial to both macrobial and microbial ecology. To do so requires microbial communities to be sampled and characterized in a way that maximizes alignment between the resulting measurements and the basis for macrobial hypotheses. While some differences are likely to persist—such as differences in species concepts—we suggest that relatively simple changes to the way microbial communities are sampled and characterized can greatly improve our ability to test macrobial hypotheses in microbial systems.

The purpose of this perspective article is to highlight two sets of choices available to microbial ecologists that are related to the units used to describe the number of taxa within a community and the range of environmental conditions (i.e., environmental extent) represented across microbial community samples. Our motivation is that there are specific—but poorly recognized—choices that enhance our ability to test macrobial hypotheses in microbial systems, while other—commonly made—choices fundamentally disconnect macrobial theory from microbial measurements.

**Figure 1:**
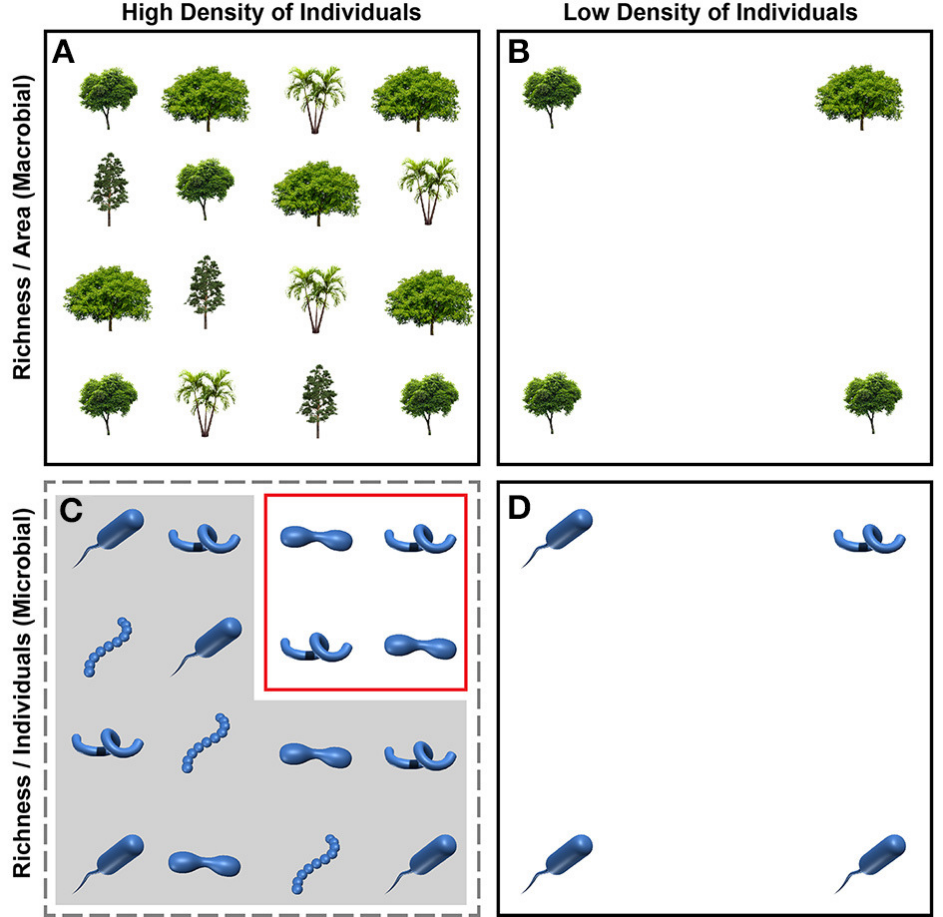
**Conceptual depiction of approaches for estimating species or OTU richness in macrobial (A,B) and microbial (C,D) ecology; each icon represents one individual**. In macrobial ecology, richness is often estimated by keeping the area sampled constant across communities **(A,B)**; richness estimates are therefore expressed as number of species per area, regardless of how many individuals are found within a given plot size. In the top panels, richness per area is 4 and 2 in the plots with a high **(A)** and low **(B)** density of individuals, respectively. In microbial ecology, OTU richness is often estimated by keeping the number of sampled individuals constant across communities **(C,D)**; richness estimates are therefore expressed as number of OTUs per individual, regardless of how the density of individuals varies across communities. Although there are more individuals per area in panel **(C)** relative to panel **(D)** most of those individuals are excluded (indicated by gray overlay) in the estimation of OTU richness; only the individuals in the red box are used in the richness estimate. Richness per individual is therefore 0.5 for both high and low density plots (2 taxa per 4 individuals sampled). This difference in how richness is quantified causes disconnect between patterns observed in microbial systems and theory developed in macrobial ecology.

In most studies to date, microbial diversity patterns and macrobial theory are decoupled because they use different units to describe the number of taxa in a given sample (Figure 1). In microbial systems the number of taxa—operational taxonomic units (OTUs)—in a given sample provides an estimate of diversity, referred to as “OTU richness.” Estimates of OTU richness are often compared across samples after standardizing the number of sequences within each community (e.g., Fierer et al., 2011; Sharp et al., 2014; Tardy et al., 2015; Zhang et al., 2015). Standardizing by number of sequences—a proxy for individuals—causes richness units to be number of OTUs per number of sampled individuals (R_indiv_) (also see Olszewski, 2004). This contrasts with richness estimates in macrobial communities, where species richness is measured per-area (e.g., Currie et al., 2004; Kraft et al., 2011; Jetz et al., 2012). Macrobial theory has therefore been developed to explain species per-area. Species-energy theory, for example, links energy per-area to species per-area (Wright, 1983; Hurlbert and Stegen, 2014).

In addition to the units of OTU richness, it is important to consider the environmental extent across which OTU richness is estimated. Hypothesis evaluation in many microbial diversity studies proceeds by finding environmental variables that explain variation in R_indiv_ without considering environmental extent. While rarely examined or accounted for in microbial ecology, environmental extent can strongly impact the outcome of statistical analysis (Rahbek, 2005; Soininen et al., 2009; Sandel and Corbin, 2010; Sandel, 2015), and may therefore influence our understanding of what governs OTU richness. For example, sampling a broad pH range and a narrow % carbon range may increase the probability of pH being inferred as the dominant driver. As such, the explicit consideration of environmental extent is a critical issue that—despite its importance—has been largely neglected in microbial ecology.

Here we use data from a field study of soil microbial communities to (*i*) provide a new sampling approach that quantifies OTU richness in a manner analogous to species-per-area, (*ii*) show that inferred drivers of OTU richness depend critically on environmental extent, and (*iii*) illustrate how researchers can generate additional insights into processes influencing OTU richness by combining sampling methods and studying patterns that emerge when environmental extent is restricted.

## Materials and methods

### Study site and field methods

The field site (Supplementary Figure 1) is located within the Caribou-Poker Creek Research Watershed (CPCRW), which comprises a relatively pristine, ~100 km^2^ watershed ~45 km Northeast of Fairbanks, AK. The CPCRW is part of the Bonanza Creek Long-Term Ecological Research site (http://www.lter.uaf.edu/). In September 2013 twelve plots were established along a West-facing hill slope (Supplementary Figure 1) characterized by spatially continuous black spruce and underlain by permafrost. Four transects were established and each contained three plots across the hill-slope. Plots were located so that each transect spanned gradients in active layer depth and tree stand structure (data not shown). Plot elevations were not consistent across transects, so hill-slope positions are referred to as low, middle, and high (Supplementary Figure 1).

At two representative locations within each plot, 7.5 cm diameter soil cores were collected to capture both organic and mineral soil layers. Cores were collected using a soil recovery augur (AMS, Inc., American Falls, ID) whereby soil samples were automatically collected into plastic sleeves. Samples were capped and frozen on dry ice within 12 h of collection and stored at −80°C until processing.

### Soil core processing

In the laboratory, the soil core outer surface was allowed to thaw to the point that core material could be pushed partially out of the plastic sleeve. Once exposed, the outer ~1 cm of soil material was removed to minimize contamination. For all steps of soil core processing, materials contacting soil were heat-treated at 450°C for 8 h to remove contamination. Visual evaluation of the still-frozen soil was used to identify core sections that were predominantly organic or mineral soils. Due to soil compression and the need to maintain the frozen state of the samples, we did not attempt to formally assign soil horizons. Instead, we focused on collecting clearly differentiated soil types from each core, with the purpose of maximizing the range of sampled conditions. Within each core, two sections were collected (each ~3.5 cm in length), one from a predominantly organic section and one from a predominantly mineral section. While still frozen each section was further fractionated into three samples that were returned to −80°C prior to further processing. Afterwards, individual samples were thawed and homogenized. To avoid potential biological and chemical changes during processing (such as those caused by drying and sieving soil samples), visible roots and rocks were manually removed using sterile forceps. Soil pH was estimated using a Denver Instrument model 215 pH ATC electrode within a 1:1 slurry of soil and 0.01 M CaCl_2_; pH ranged from 3.11 to 6.42 across samples.

DNA was isolated from 0.25 g (wet mass) of soil from each sample using the MoBio PowerSoil kit following manufacturer's instructions, but with the addition of a 2-h proteinase-K incubation at 55°C prior to the bead-beating step to facilitate cell lysis. DNA concentrations were estimated using PicoGreen, and PCR reactions and sequencing were assumed to indicate sufficient DNA quality. Quantitative PCR (qPCR) was used with the DNA extracts to estimate bacterial 16S rRNA gene-copy-number, as a proxy for individual bacterial cells g^−1^ of soil. This analysis used the same DNA extraction as the 16S rRNA gene sequencing (see below), but was otherwise completely independent of the sequencing effort. qPCRs were carried out at the DNA Services Facility at the University of Illinois, as follows. Quantification of bacterial small subunit rRNA genes (SSU rRNA) was performed as described previously by Nadkarni et al. (2002), using Taqman 2X Gene Expression Master Mix (Invitrogen, Foster City, CA). Primers and probe were obtained from Integrated DNA Technologies (IDT; Coralville, IA). Absolute quantification was performed using a standard curve derived from PCR products generated by near-full gene amplification of SSU rRNA genes using the general bacterial primer set 27F and 1492R (Lane, 1991). The standard curve was linear across five orders of magnitude (from 2.44E+06 to 2.44E+01 copies/reaction), with a 99% efficiency. Three technical replicates were run for each sample, and analyzed using the ABI ViiA7 RealTime PCR system. Across samples, 16S rRNA gene-copy-numbers ranged from 4.54 × 10^5^ to 1.88 × 10^10^ g^−1^ (soil dry mass).

To characterize bacterial communities, DNA extracts were used to amplify the V4 region of the 16S rRNA gene using the 515F/806R primer set with triplicate PCRs, which were pooled for each sample. Normalized concentrations of PCR products were sequenced on an Illumina MiSeq at Argonne National Laboratory using 250 × 250 paired-end chemistry. Raw sequences were processed using QIIME: paired-end sequences were joined and demultiplexed, chimeras were identified and removed using USEARCH 6.1 within QIIME, sequences were clustered into operational taxonomic units (OTUs) based on 97% sequence similarity using USEARCH 6.1 (within QIIME) with open reference OTU picking and the SILVA database as the reference.

### Data analysis

Data analysis was carried out in R version 3.2.1 (R-Core-Team, 2015). Preliminary data evaluation revealed that some variables were characterized by skewed distributions (particularly gene copy density) and potential outliers. All variables were therefore log_10_-transformed to improve normality. A few remaining outlier data points, following transformation, were removed prior to linear regression analyses (Supplementary Figure 2). All remaining analyses were carried out using linear regression. Transforming no variables or only log_10_-transforming gene copy density did not qualitatively alter the results (Supplementary Figures 3, 4).

**Figure 2:**
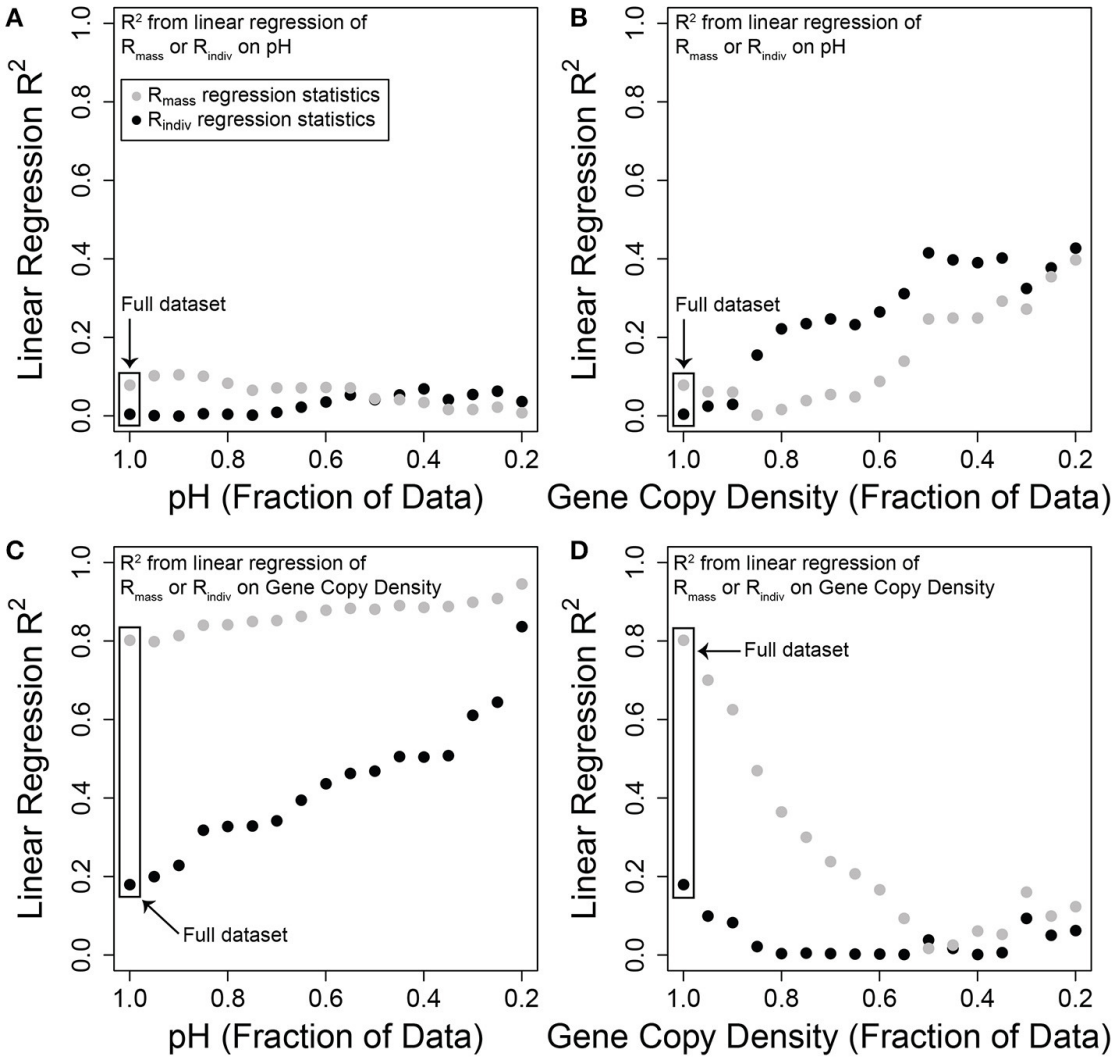
**Variation in R_**mass**_ and R_**indiv**_ explained by either pH or gene-copy-density, using different subsets of each explanatory variable**. *R*^2^ values are derived from linear regression models using log-transformed variables. The *x*-axis in each panel indicates the fraction of data used in the linear regression. For example, the fraction of 1 in panel **(A)** indicates that the full dataset was used to characterize the linear relationship between pH and either R_mass_ (gray) or R_indiv_ (black); the plotted points indicate the *R*^2^ values of those linear regressions. A fraction of 0.2 indicates that only samples in the upper 20% of the pH distribution were used to characterize these linear relationships. In turn, moving from left to right within panel **(A)** the sample set used in each linear regression includes a smaller range of pH values. **(B)** Regression statistics for the linear relationship between pH and either R_mass_ or R_indiv_, across different fractions of the gene-copy-density distribution. **(C)** Regression statistics for the linear relationship between gene-copy-density and either R_mass_ or R_indiv_, across different fractions of the pH distribution. **(D)** Regression statistics for the linear relationship between gene-copy-density and either R_mass_ or R_indiv_, across different fractions of the gene-copy-density distribution.

To examine the influence of changing the sampled environmental extent, we ran linear regression models across the full dataset and for subsets of the dataset. For each explanatory variable we dropped samples falling below a given value and then used the resulting (i.e., reduced) dataset to re-estimate the variation in OTU richness explained by pH or gene-copy-density. The threshold was then increased, samples falling below the new threshold were dropped, and variation explained by pH or gene-copy-density was re-estimated. This procedure was repeated for each explanatory variable until only samples in the top 20% of the distribution (e.g., the top 20% of the pH distribution) were retained. The analysis was not conducted beyond a threshold of the top 20% due to a small number of data points remaining when using smaller portions of the data (also see Figure 2).

To illustrate that there is resonance between our microbial results and patterns observed in macrobial systems, we also estimated species richness using data on North American breeding birds. As with our microbial analyses, these data come from only one system and are therefore considered an illustrative example. The associated dataset has been used previously to study hypotheses related to species richness gradients, and includes data necessary for estimating species richness per-individual and per-area. Data were from the year 2003 from the subset of 435 North American Breeding Bird Survey routes used in Coyle et al. (2013), where each route covers 25 km^2^. Data from other years showed similar patterns. Data were log_10_ transformed for consistency with the OTU richness analyses.

## Richness estimation and hypothesis development

OTU richness was estimated using two sampling approaches: (*i*) by rarefying all communities to a consistent number (3504) of sequences and counting the number of unique OTUs in each sample (R_indiv_), which resulted in dropping 12 samples out of 165 due to those samples having fewer than 3504 sequences, and (*ii*) per-mass of soil by allowing the number of sampled sequences to vary across communities in proportion to the number of 16S rRNA gene copies g^−1^ (proxy for individuals per g of soil), and then counting the number of unique OTUs in each sample (R_mass_). R_mass_ holds the soil mass per community constant, while R_indiv_ holds the number of sampled sequences (individuals) constant.

For R_mass_, a community with 10× higher gene-copy-density would ideally be characterized by sampling 10× more sequences. However, in our dataset, this required some communities to contain more sequences than were available. There are many potential solutions to this problem, and as a starting point we used log-based proportionalities. The number of sequences used to characterize a given community (*S*_samp_) was calculated as *S*_*samp*_ = *S*_*min*_*Log*_10_(10 · *G*_*samp*_/*G*_*min*_), where *S*_*min*_ is the absolute number of sequences used to characterize the community that had the lowest gene-copy-density, *G*_*min*_ is the lowest gene-copy-density found across all samples, and *G*_*samp*_ is the gene-copy-density of the community being characterized. A community with 10× or 100× higher gene-copy-density therefore received 2× or 3× more sequences, respectively.

In the North American Breeding Bird dataset, we estimated R_indiv_ and species richness per-area (R_area_), the latter as an analog of R_mass_. To estimate R_indiv_ each survey route was rarefied to 84 individuals, which was the lowest number of individuals observed across routes. The number of species observed in a given route, after sampling 84 individuals, was used as an estimate of R_indiv_. R_area_ was estimated as the total number of species observed within each survey route, regardless of how many total individuals were observed.

From species-energy theory we hypothesize that R_mass_ will be best explained by gene-copy-density; more individuals g^−1^ can support more species g^−1^ (Wright, 1983; Hurlbert and Stegen, 2014). Niche-conservatism (Wiens et al., 2010) provides an alternative hypothesis: R_mass_ will be best explained by pH due to specialized physiological mechanisms required to maintain intracellular pH at extreme pH (Slonczewski et al., 2009). In this case R_mass_ would be governed by the number of OTUs capable of persisting at a given pH. We further hypothesize that support for species-energy theory or niche-conservatism will diminish as the range of sampled gene-copy-densities or pH is decreased, respectively.

There is no inherent connection between species-energy theory and R_indiv_ because information related to individuals g^−1^ is removed during R_indiv_ estimation. Variation in R_indiv_ is driven instead by changes in the shape of the species abundance distribution (SAD) (also see Olszewski, 2004). At one extreme, R_indiv_ is minimized when there are a small number of dominant taxa (i.e., a strongly skewed SAD). This occurs because a discrete number of individuals is used to describe the community and most of those individuals will belong to a small number of dominant taxa; the result is low R_indiv_. At the other extreme, R_indiv_ is maximized when all taxa are equally abundant (i.e., a flat SAD). This occurs because each taxon has the same chance of being observed such that sampled individuals are broadly distributed across taxa; the result is high R_indiv_. In addition, there is no obvious link between the shape of SAD and the niche-conservatism hypothesis. We therefore use the balance of empirical evidence in soil systems to hypothesize that R_indiv_ will be best explained by pH (Fierer and Jackson, 2006; Chu et al., 2010; but see Lipson et al., 2015). We further hypothesize that variation in R_indiv_ explained by pH or gene-copy-density will decrease with decreases in the sampled ranges of these variables.

## Results and discussion

Variation in R_indiv_ and R_mass_ explained by pH and by gene-copy-density across different subsets of the microbial dataset is summarized in Figure 2. Using the whole dataset, R_indiv_ and R_mass_ were both best explained by gene-copy-density (c.f. Figures 2A,C), contrary to our hypotheses. Variation explained by gene-copy-density was, however, greater for R_mass_ (Figure 2C), which we also observed in the macroecological dataset (Supplementary Figure 5). Taking gene-copy-density as a proxy for individuals g^−1^ therefore provides support for species-energy theory in terms of more individuals leading to higher R_mass_. To fully link our analyses to species-energy theory would, however, require knowledge and quantification of limiting resource supply; at present we assume that greater resource supply leads to more individuals g^−1^. Interestingly, the increase in R_indiv_ with gene-copy-density suggests factors—such as habitat heterogeneity—that promote more even abundance distributions may also increase with resource availability, a pattern observed in macroecological systems (Hurlbert, 2004; Hurlbert and Jetz, 2010). Therefore, OTU richness is not driven purely by number of individuals g^−1^ (Hurlbert and Jetz, 2010); conditions that lead to more individuals g^−1^ also lead to flatter SADs and, in turn, higher R_indiv_.

Consistent with our hypotheses, reducing the range of sampled pH increased the variation in both richness estimates explained by gene-copy-density. This effect was dramatic for R_indiv_, but modest for R_mass_ (Figure 2C). Sampling a broader range of pH may therefore partially mask the influence of selective pressures associated with low gene-copy-density that lead to increased dominance by a few taxa.

Also consistent with our hypotheses, reducing the range of sampled gene-copy-densities caused declines in R_indiv_ and R_mass_ variation explained by gene-copy-density. In particular, removing 20 or 50% of the gene-copy-density distribution resulted in gene-copy-density explaining almost no variation, respectively, in R_indiv_ or R_mass_ (Figure 2D), but increased variation in R_indiv_ and R_mass_ explained by pH (Figure 2B). For every 10% of the gene-copy-density distribution that was not sampled, there was a decrease of ~16% in R_mass_ variation explained (Figure 2D). The inference of a pH-driver and support for species-energy theory are, therefore, clearly impacted by environmental extent.

We note that the details of how explained variation changes with environmental extent will depend on how data are systematically removed. We elected to remove data from the low-end of pH and gene-copy-density ranges, and we consider this an illustrative starting point rather than a definitive approach, as other methods could be applied. In particular, data could be removed from the high-end of each variable or simultaneously from each end. We encourage further study of how these different approaches may yield additional insights.

We have shown here that units associated with OTU richness and sampled environmental extent constrain which theoretical frameworks can be evaluated. On the other hand, recognizing these constraints provides an opportunity to take advantage of them to derive additional insights into the mechanisms governing microbial diversity. Below we provide an example of such an approach using the combination of R_mass_ and R_indiv_ patterns observed in our study system.

R_mass_ has a clear conceptual linkage to species-energy theory and we find strong support for the underlying hypothesis that more individuals—per mass of soil—allow more OTUs to coexist, consistent with recent work in microbial systems (Locey and Lennon, 2016). We therefore infer that limiting resource supply is likely the dominant, albeit indirect, driver of OTU richness in our study system. On the other hand, R_indiv_ controls for variation in individuals g^−1^ and therefore removes the dominant influence of resource supply. This provides an opportunity to gain insight into additional factors that influence OTU richness, as discussed below.

While we found that R_indiv_ was also related to the number of individuals g^−1^, it is critical to recognize that a relationship between R_mass_ and individuals g^−1^ has a fundamentally different interpretation than a relationship between R_indiv_ and individuals g^−1^; R_mass_ increasing with individuals g^−1^ is consistent with species-energy theory, whereas R_indiv_ increasing with individuals g^−1^ is not conceptually related to species-energy theory. Instead, R_indiv_ increasing with individuals g^−1^ points to other mechanisms such as habitat heterogeneity. In turn, one interpretation of our data is that OTU richness is governed by a combination of resource supply—at the scale of the measured soil volume—and niche partitioning within the spatially complex soil volume. This inference would not be possible without combining the two sampling strategies that generate R_mass_ and R_indiv_ and carefully considering their unique properties.

Restricting the range of gene-copy-densities provides another example of how additional insights can be gained by taking advantage of constraints imposed by units and environmental extent. In our system, restricting the range of gene-copy-densities flipped the relative explanatory power of pH and gene-copy-density for both R_mass_ and R_indiv_. This is instructive as it points to yet another set of mechanisms that combine with bulk resource supply and habitat heterogeneity to govern OTU richness. It also highlights that as the gene-copy-density extent is restricted, R_mass_ becomes equivalent to R_indiv_. This equivalency is reflected in pH explaining the same amount of variation in R_mass_ and R_indiv_ at the most restricted range of gene-copy-densities (Figure 2B). In turn, given a small range of gene-copy-densities, variation in both R_mass_ and R_indiv_ is driven by changes in the shape of the SAD. Following that observation—and assuming that a small range in gene-copy-densities reflects little variation in bulk resource supply—one could infer that under a scenario of homogeneous resource supply, higher pH leads to a flatter SAD and therefore higher OTU richness. This interpretation suggests strong selection at low pH whereby a small number of OTUs are dominant under low pH conditions, leading to a more peaked SAD. While not a strong test, this is conceptually consistent with the niche conservatism hypothesis. When combined with inferences discussed above, this suggests that OTU richness is governed by a combination of mechanisms that underlie species-energy theory and the niche conservatism hypothesis.

In summary, we have provided a new sampling approach that allows OTU richness to be quantified in a way that aligns with macroecological theory and have highlighted the fact that while R_indiv_ is commonly estimated, it is fundamentally decoupled from macroecological theory. We also illustrated how combining sampling strategies and manipulating environmental extent can provide additional insight—relative to using a single sampling strategy or environmental extent—into factors governing microbial diversity. While the approaches used here do not solve all discrepancies between microbial and macrobial richness estimation, we suggest that embracing them will enhance the ability of microbial ecologists to test and contribute to macroecological theory.

## Author contributions

JS, AH, BL, XC, and NH designed research; JS, BL, XC, RC, SF, and MT performed research; JS, AH, BL, XC, CA, RC, FD, SF, NH, and MT contributed to concepts and writing contained in the paper.

### Conflict of interest statement

The authors declare that the research was conducted in the absence of any commercial or financial relationships that could be construed as a potential conflict of interest.
