# Supplementary material for: Aligning the Measurement of Microbial Diversity with Macroecological Theory
Source: Front Microbiol. 2016 Sep 23;7:1487. doi: 10.3389/fmicb.2016.01487 (PMC5033968; doi:10.3389/fmicb.2016.01487)
Supplement: Supplementary file 1 [file Presentation1.PDF]

Supplementary Material

Aligning the Measurement of Microbial Diversity with Macroecological Theory

James C. Stegen[*1], Allen H. Hurlbert[2], Ben Bond-Lamberty[3], Xingyuan Chen[4], Carolyn G. Anderson[1], Rosalie K. Chu[5], Francisco Dini-Andreote[6], Sarah J. Fansler[1], Nancy J. Hess[5], and Malak Tfaily[5]

*** Correspondence:** James C. Stegen James.Stegen@pnnl.gov

1 Supplementary Figures

1.1 Supplementary Figures

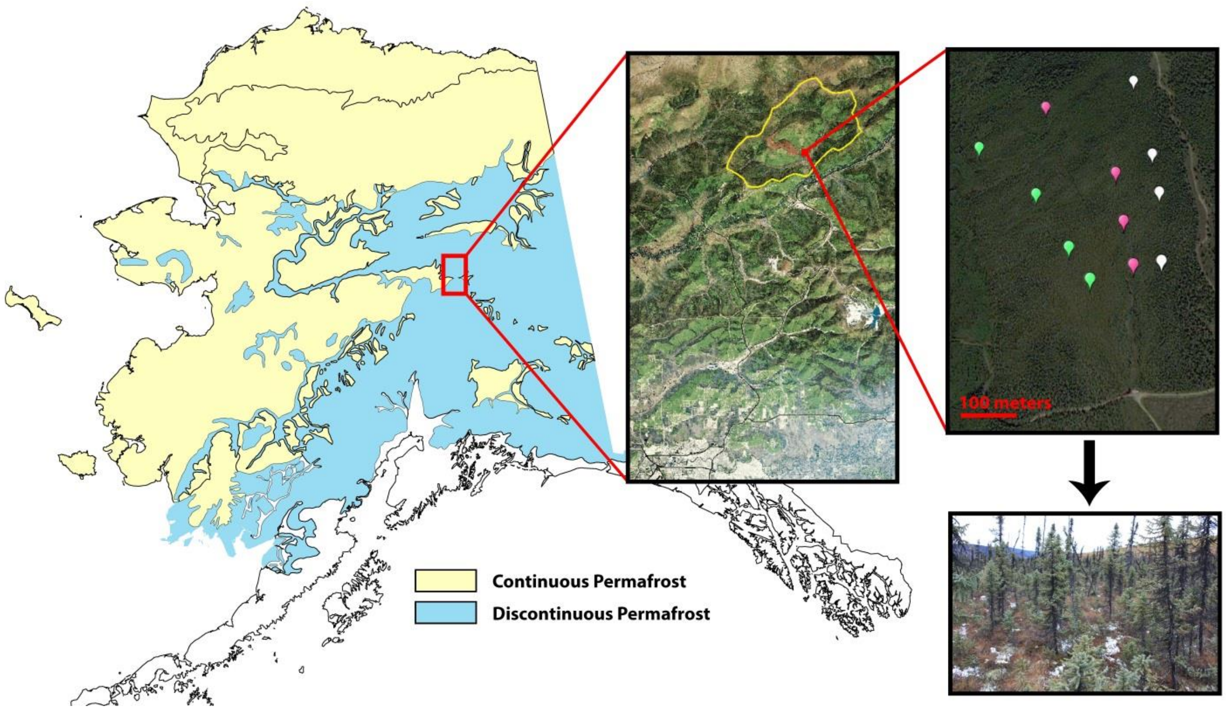

Supplementary Figure 1. Spatial layout of field sampling locations. The left panel shows Alaska with estimated permafrost distributions. The middle panel shows the Caribou Poker Creeks Research Watershed boundary (yellow polygon). The upper right image shows the spatial layout of sampling plots; white, pink and green icons respectively indicate high, middle, and low elevations within each of the four transects. The lower right panel is an image from the field site showing a representative black spruce stand.

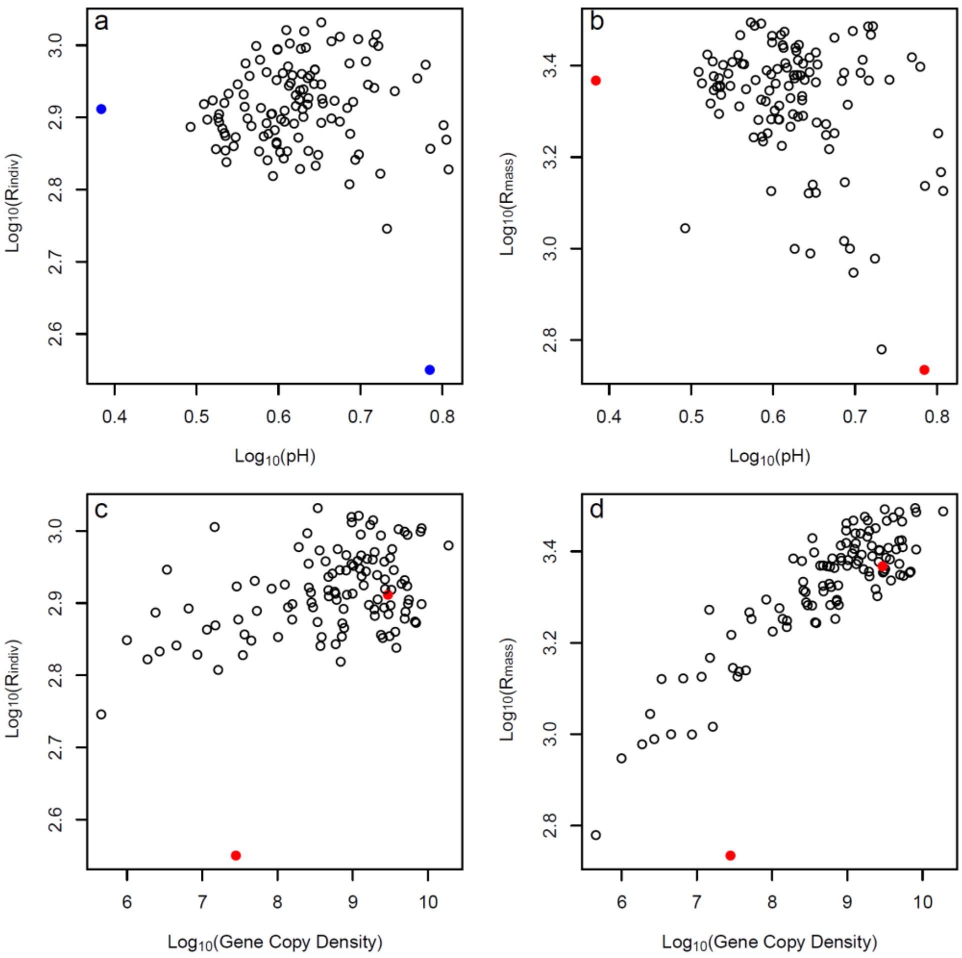

Supplementary Figure 2. Bivariate plots relating OTU richness estimates to pH and 16S rRNA gene-copy-density (per gram of soil); all axes show Log_{10} transformed values. Open circles in all panels were used in the analyses that generated Figure 2. (a) Solid blue circles indicate data points that were considered outliers and were removed prior to analysis. Samples containing these outlier data points were completely removed from the analysis. This resulted in data points being dropped from the other panels; the dropped data points are shown as solid red circles (b-d).

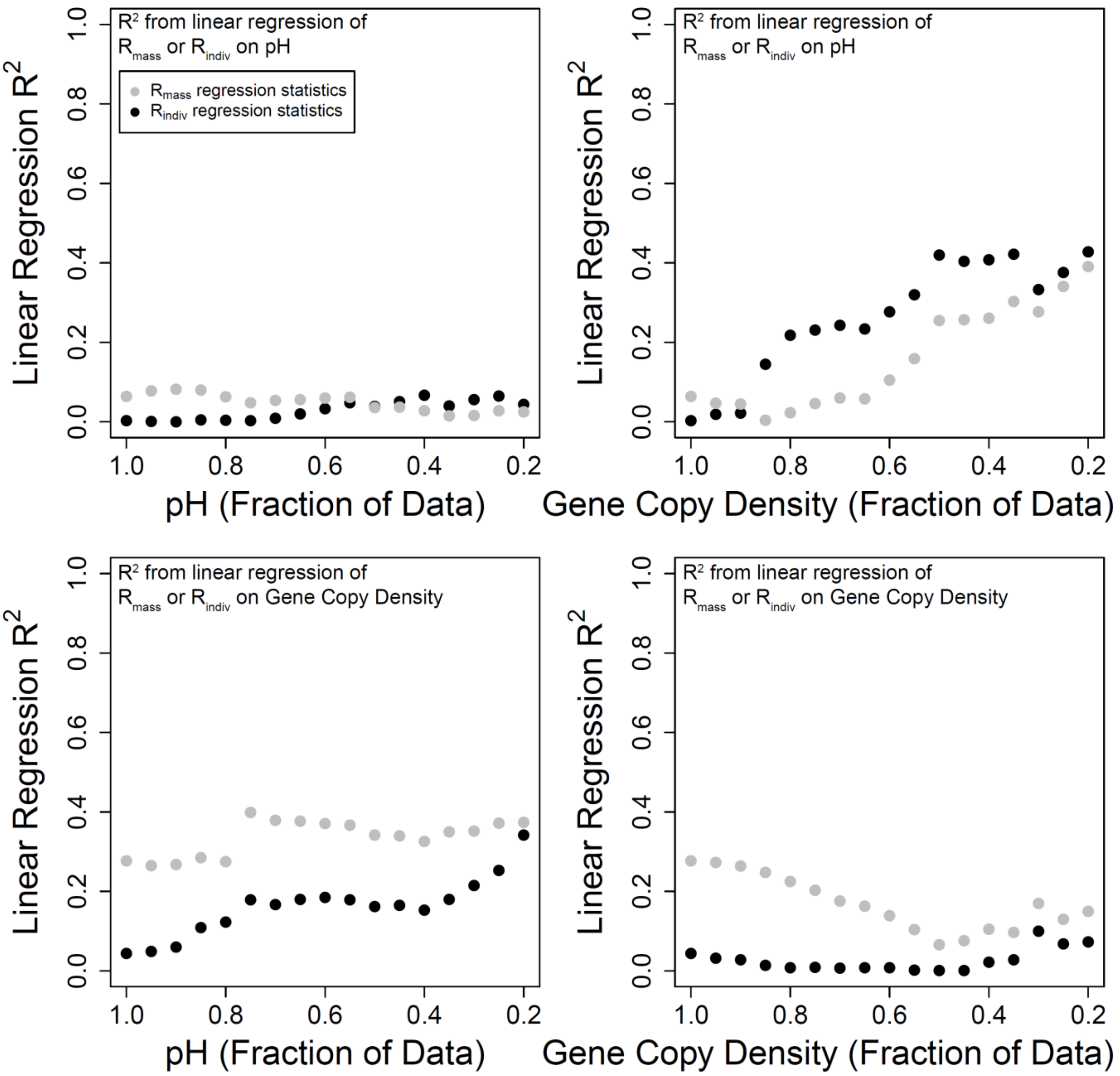

Supplementary Figure 3. Variation in richness metrics explained by either pH or gene-copy-density, using different subsets of each explanatory variable; R^2 values are derived from linear regression models using log-transformed variables. Data were not transformed prior to analysis. All other details as in Figure 2 in the main manuscript.

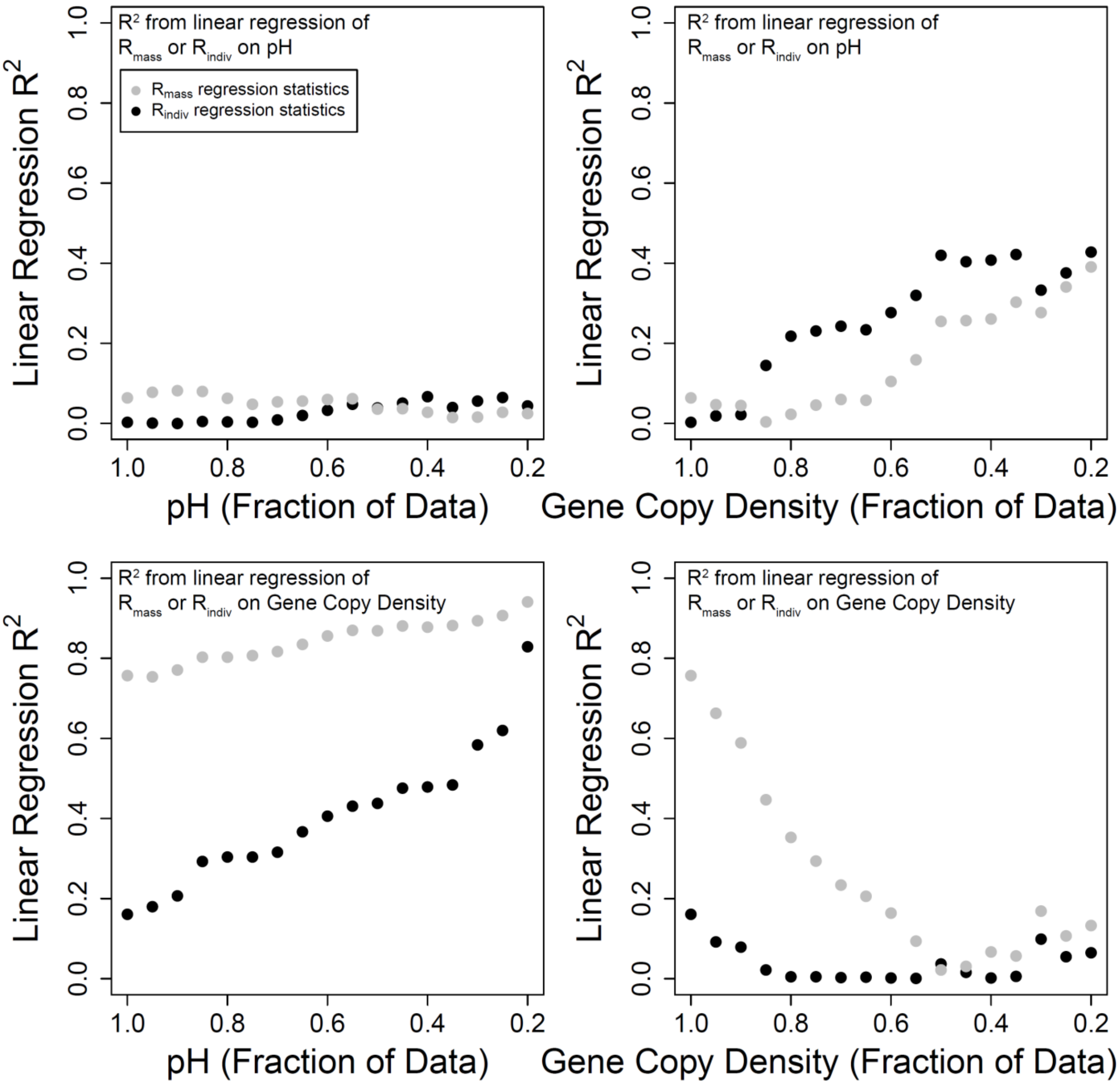

Supplementary Figure 4. Variation in richness metrics explained by either pH or gene-copy-density, using different subsets of each explanatory variable; R^2 values are derived from linear regression models using log-transformed variables. Only gene-copy-density data were $\log_{10}$-transformed prior to analysis. All other details as in Figure 2 in the main manuscript.

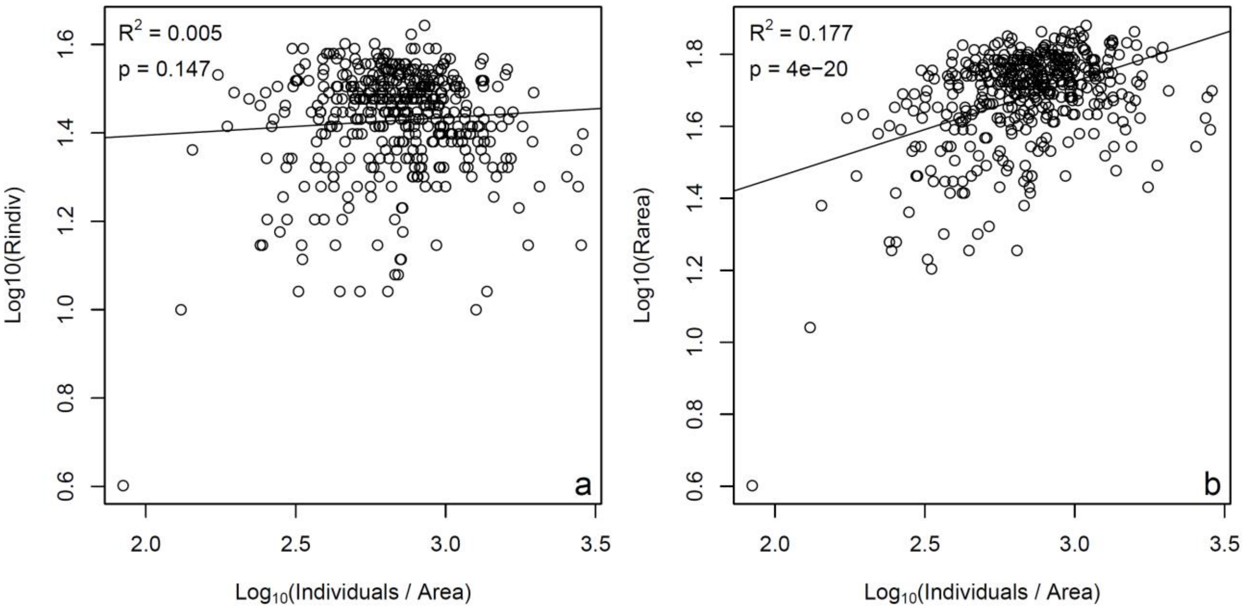

Supplementary Figure 5. Species richness related to individual density in North American breeding birds. Solid lines represent linear regression models. (a) Species richness per individual (R_{indiv}) was not related to individuals per area (p = 0.147). (b) Species richness per area (R_{area}) increased with individuals per area ($p = 4 \times 10^{-20}$).
